# Cardiac magnetic resonance assessment of aortic distensibility in prediabetic patients

**DOI:** 10.1186/s43044-020-0040-0

**Published:** 2020-01-21

**Authors:** Nady A. Razik, Y. T. Kishk, Madeeha Younis Bakheet, Mina Nous, Mohamed Abdel Ghany

**Affiliations:** Assiut University, Asyut, Egypt

**Keywords:** Aortic distensibility, Prediabetes, Metabolic syndrome, CMR, CAD

## Abstract

**Background:**

Hyperglycemia, insulin resistance, and hyperinsulinemia represent important pathophysiological components of the prediabetic stage that result in arteriosclerosis and increased arterial stiffness. We sought to compare the aortic distensibility (AD) assessed by cardiac magnetic resonance (CMR) in prediabetic patients presenting with chronic coronary artery disease (CCAD) versus patients with normal HbA1C. Ninety-eight patients with CCAD were recruited. All patients were screened for HbA1C levels and then underwent a CMR study to assess AD of the aortic root and the ascending and descending thoracic aorta. Patients were classified into two groups: 52 prediabetic (HbA1C 5.7–6.4%) (*study group*) and 46 with normal glycemic status (HbA1C < 5.7%) (*control group*).

**Results:**

AD values at the aortic root (AR) (13.93 ± 5.17 vs 34.3 ± 9.65 Kpa^-1^ × 10^-3^), ascending aorta (AA) (13.17 ± 4.81 vs 28.1 ± 8.33 Kpa^-1^ × 10^-3^), and descending thoracic aorta (DA) (18.12 ± 4.34 vs 33.68 ± 7.57 Kpa^-1^ × 10^-3^) were significantly lower in the study group than in the control group (*P value for all was* < 0.001). Twenty-eight patients fulfilled the criteria for metabolic syndrome, and in those patients, AD was significantly lower than in those without metabolic syndrome.

Aortic distensibility at the AR, AA, and DA had strong significant negative correlations with the level of glycosylated hemoglobin (AA, AR, DA; *r* − 0.66, − 0.68, − 0.58, respectively) *(P <* 0.001).

**Conclusion:**

AD values at different points (AR, AA, and DA) were significantly lower in prediabetic and metabolic syndrome patients than in controls. These values also showed a significant negative correlation with the levels of HBA1C.

## Background

Arterial stiffness is an early detectable manifestation of adverse structural and functional changes of the vessel wall [1].

Insulin resistance can promote arterial stiffness and plaque progression through downregulation of insulin signaling pathways and alterations in lipid metabolism; moreover, inflammatory pathways support the role of insulin resistance in the pathophysiology of aortic stiffness [2].

Increased arterial stiffness is an early phenomenon that occurs in the impaired glucose metabolic state [3]. Insulin has acute vasodilator effects that lead to increased arterial distensibility, and this beneficial effect is blunted in insulin-resistant states. Increased stiffness is a feature of insulin resistance [4].

Chronic hyperglycemia and hyperinsulinemia increase the local activity of the renin-angiotensin aldosterone system and expression of angiotensin type I receptor in vascular tissue, promoting the development of wall hypertrophy and fibrosis [5].

In addition, low-grade inflammation and endothelial dysfunction, which are interrelated, may also explain, at least in part, the increase in arterial stiffness related to diabetes and metabolic syndrome [6].

Greater arterial stiffness increases cardiovascular disease risk by increasing blood pressure, increasing ventricular hypertrophy, decreasing coronary perfusion, and increasing the risk of stroke [7].

Many methods have been used to assess aortic distensibility (AD), but CMR has the unique ability to provide both local and regional direct noninvasive measures of aortic function. Lumen area changes (aortic strain) over the cardiac cycle can be acquired with high temporal and spatial resolution, and aortic distensibility can be measured in different locations [8].

## Methods

### Study subjects

Ninety-eight patients were enrolled: 8 women and 90 men diagnosed with CCAD (patients with documented CAD and stable for 3 months) underwent CMR. The study was approved by our local ethics committee, and written consent was obtained from each patient. The patients were classified into 52 prediabetics with elevated HbA1C (defined range 5.7–6.4%) (study group) and 46 patients with normal HbA1C (control group). The exclusion criteria included age < 18 years, any contraindications to MRI, and a history of DM, HTN, renal impairment, LVEF less than 45%, and aortic diseases (more than moderate aortic regurgitation; more than mild aortic stenosis and aortic aneurysm).

### Laboratory investigations

Glycosylated hemoglobin (HbA1C) for all patients was analyzed by radioimmunoassays by COBAS INTEGRA 400 PLUS/800 analyzers (Roche, USA) through the Assiut University laboratory. Complete blood count (CBC), serum urea and creatinine, and a lipid profile including total cholesterol (TC), triglycerides (TG), HDL-c (high-density lipoprotein cholesterol), and LDL-c (low-density lipoprotein cholesterol) for all patients were analyzed by Hitachi Cobas c 501 analyzers.

### Echocardiography

All patients underwent echocardiography. The same operator and same device (GE Vivid S5) was used to minimize interobservational variability. LV volumes were measured using 2D echocardiography. Significant aortic regurge was excluded by color Doppler study of the aorta in the parasternal long-axis (PLAX) and apical 5-chamber views. Aortic diameters were measured and included the aortic root, ascending aorta in PLAX view, arch, and descending aorta in suprasternal view.

### Assessment of aortic distensibility by CMR

CMR acquisition was performed with Ingenia Philips 1.5 Tesla MRI scanner. Electrocardiogram (ECG)-gated gradient-echo phase-contrast cine sequence with velocity encoding FFE with breath-holding was taken at 2 levels: one in the axial plane at the level of the sinotubular junction for calculation of aortic root luminal diameter (by the average of the three end-diastolic cusp-commissure measurements) and another perpendicular to the descending aorta at the level of the bifurcation of the pulmonary artery for calculation of ascending and descending aorta luminal diameters, with a repetition time equal to the RR interval (in ms) and a 14-ms echo time, 256 × 256 matrix size, 300-mm field of view, 8-mm slice thickness, and 20° flip angle. Acquisition was performed using two views for appropriate alignment of the cut (for example, using an LVOT view and a 3-chamber view for the aortic root and using a coronal view and LVOT view for the ascending aorta). Modulus images of the cine phase-contrast sequences were used to calculate the corresponding aortic section luminal diameter. Aortic contours were automatically traced throughout the cardiac cycle and then manually corrected (if needed) using QFLOW software (Phillips, the Netherlands).

**Fig. 1:**
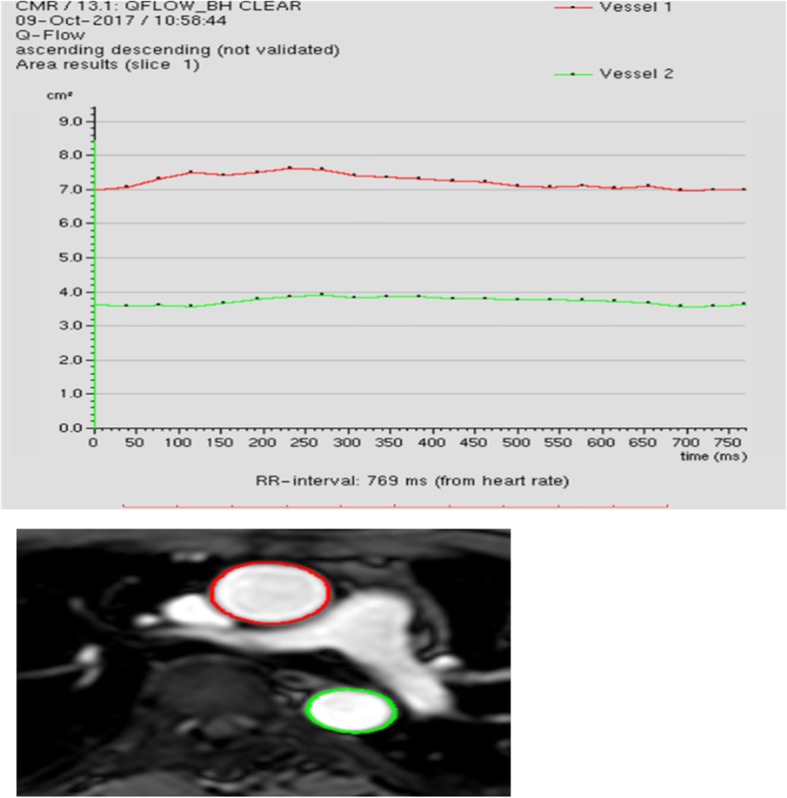
Snapshot image from Philips Q flow software. TOP: chart illustrating cross-sectional area change of ascending and descending aorta at the level of pulmonary artery bifurcation during the cardiac cycle. Bottom A modulus image of Q-flow sequence of ascending and descending aorta at the level of pulmonary artery bifurcation with contouring

The maximum and minimum ascending and descending aortic luminal areas were determined from the maximum and minimum cross-sectional areas of the corresponding aortic section, respectively, and then, we obtained a curve and generated a table (plotting the luminal area against trigger time) (Fig. 1). Using this curve, we measured the aortic distensibility of the corresponding aortic section with the following equation: 1000× (systolic aortic area (cm^2^) − diastolic aortic area (cm^2^)/(diastolic aortic area (cm^2^) × pulse pressure (mmHg) [8].Systolicaorticareacm^2^-Diastolicaorticareacm^2^*Distolicaorticarea*cm^2^xPulsepressure(mmHg)

Blood pressure (BP) was measured twice at the time of the scan, and the average reading was used. Pulse pressure was calculated as the difference between systolic and diastolic BP.

## Results

### Patient characteristics

The mean age of the patients was 51.04 ± 6.44 years, and the majority of them were males (92.3%), while the mean age of the controls was 49.30 ± 6.31 years, and the majority (91.3%) of them were males. The mean body mass index was significantly higher in the patients with prediabetes (30.07 ± 4.76 kg/m^2^) than in the controls (25.62 ± 3.02, *P* < 0.001). Additionally, the patients with prediabetes had significantly higher waist circumference than the controls (108.5 ± 7.13 cm versus 93.70 ± 7.70 cm; *P* < 0.001).

The patients with prediabetics had significantly higher diastolic blood pressure and pulse pressure than the controls (*P* < 0.001), but systolic blood pressure was not significantly different between the two groups (*P* = 0.91) (Table 1).

**Table 1:** Clinical characteristics of the study and control groups

Patient characteristics	Study group	Control group	*P* value
Age (years)	51.04 ± 6.44	49.30 ± 6.31	0.34
BMI	30.07 ± 4.76	25.62 ± 3.02	< 0.001
Waist circumference (cm)	108.5 ± 7.13	93.70 ± 7.70 cm	< 0.001
SBP	118.46 ± 5.24	118.68 ± 4.56	0.91
DBP	76.92 ± 7.35	82.60 ± 4.48	< 0.001
PP	41.53 ± 5.43	36.08 ± 4.99	< 0.001
Lipid profile			
Glycosylated hemoglobin (Hgb A1c)	6.02 ± 0.23	5.22 ± 0.41	< 0.001
Cholesterol (mg/dl)	200.11 ± 39.37	183.20 ± 40.30	0.22
Triglyceride (mg/dl)	157.19 ± 41.60	144.13 ± 29.88	0.29
HDL (mg/dl)	37.73 ± 6.78	45.80 ± 4.69	< 0.001
LDL (mg/dl)	130.80 ± 28.40	99 ± 19.17	< 0.001
Echo parameters			
Ejection fraction (%)	53.23 ± 5.07	55.80 ± 2.04	0.09
Aortic root (cm)	3.16 ± 0.41	3.08 ± 0.35	0.45
Left atrium (cm)	3.98 ± 0.6	4.28 ± 0.70	0.13

There were no significant differences between the two groups regarding aortic root diameter, aortic velocity index, left atrium diameter, and ejection fraction (Table 1).

Hemoglobin and creatinine levels were not significantly different between the two groups (*P* > 0.05). The study showed that cholesterol (200.11 ± 39.37 versus 183.20 ± 40.30, *P* = 0.22) and triglyceride (157.19 ± 41.60 versus 144.13 ± 29.88, *P* = 0.29) levels were non-significantly higher in the study group than in the control group, while LDL levels were significantly higher in the study group than in the control group (130.80 ± 28.40 versus 99 ± 19.17, *P* < 0.001, respectively). HDL levels were significantly lower in the study group than in the control group (37.73 ± 6.78 versus 45.80 ± 4.69, *P* < 0.0001, respectively) (Table 1).

### Aortic distensibility by CMR

Aortic distensibility values at different points (aortic root, ascending aorta, and descending aorta) were significantly lower in the prediabetes patients than in the controls (*P* < 0.001) (Table 2). Moreover, these values had a strong significant negative correlation with the level of glycosylated hemoglobin (*P <* 0.001) (Table 3).

**Table 2:** Aortic distensibility in study and control groups

Distensibility	Study group (*n* = 52)	Control group (*n* = 46)	*P* value
AR distensibility			
Kpa^-1^ × 10^-3^	13.93 ± 5.17	34.30 ± 9.65	< 0.001
AA distensibility			
Kpa^-1^ × 10^-3^	13.17 ± 4.81	28.10 ± 8.33	< 0.001
DA distensibility			
Kpa^-1^ × 10^-3^	18.12 ± 4.34	33.08 ± 7.57	< 0.001

**Table 3:** Correlation between aortic distensibility, HbA1C, age, BMI, and waist circumference

	Aortic distensibility (kpa^-1^ × 10^-3^)					
	Aortic root		Ascending aorta		Descending aorta	
	*R*	*P* value	*r*	*P* value	*r*	*P* value
HbA1C	− 0.66	< 0.001	− 0.68	< 0.001	− 0.58	< 0.001
Age	− 0.03	0.83	− 0.40	0.03	− 0.08	0.56
BMI	− 0.43	< 0.001	− 0.38	< 0.001	− 0.41	< 0.001
Waist circumference	− 0.74	< 0.001	− 0.70	< 0.001	− 0.60	< 0.001

### Correlation between aortic distensibility and glycosylated hemoglobin

**Fig. 2:**
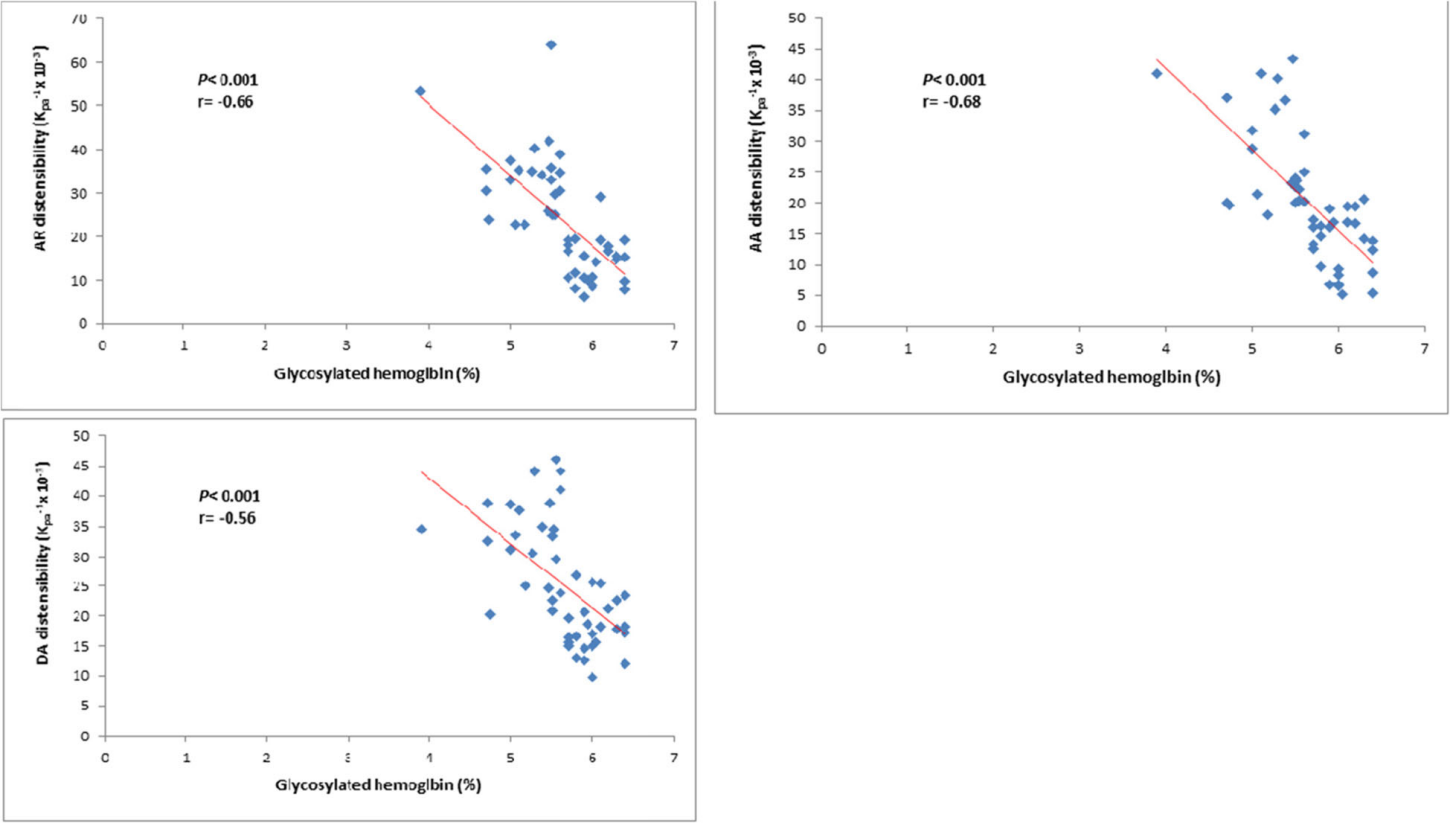
Correlation between Hba1C and aortic distensibility at different levels

Aortic distensibility values in different areas (aortic root, ascending aorta, and descending aorta) had strong significant negative correlations with the levels of glycosylated hemoglobin (*P <* 0.001) (*r* = 66, 68, 58, respectively) (Table 3, Fig. 2).

### Relationship between aortic distensibility and age

Aortic distensibility at the aortic root and descending aorta had no significant correlations with the age of the subjects (*P >* 0.05), but distensibility of the ascending aorta had a moderately significant correlation with age (*r* = − 0.40, *P* = 0.03) (Table 3).

### Correlation between aortic distensibility and body mass index

Aortic distensibility in different areas (aortic root, ascending aorta, and descending aorta) had significant negative correlations with body mass index values (*P <* 0.001) (Table 3). Moreover, aortic distensibility in different areas (aortic root, ascending aorta, and descending aorta) had significant negative correlations with waist circumference values (*P <* 0.001) (Table 3).

Using multivariate regression analysis, the following were predictors for aortic stiffness in the prediabetes patients with an adjusted *R*^2^ value of 0.59: body mass index, glycosylated Hb, and waist circumference (Table 4).

**Table 4:** Multivariate regression analysis for prediction of aortic stiffness

Variables	Odd’s ratio	95% confidence interval	*P* value
Body mass index	1.45	1.34–3.45	0.01
Glycosylated Hb	2.18	2.01–5.45	0.01
HDL	0.45	0.33–1.98	0.45
LDL	1.56	0.87–1.99	0.29
Waist circumference	1.22	1.11–2.10	0.01

### Relationship between aortic distensibility and metabolic syndrome

Twenty-eight patients were diagnosed with metabolic syndrome (25 from the study group and 3 from the normal glycemic group) based on the criteria of the modified ATP III (Adult Treatment Panel III) published by the IDF (International Diabetes Federation) in 2005 [9]. Aortic distensibility values at different points (aortic root, ascending aorta, and descending aorta) were significantly lower in those patients with metabolic syndrome than in those without metabolic syndrome (AR: 13.36 vs 17.05, *P* < 0.01; AA: 12.48 vs 16.93, *P* = 0.04; DA: 18.03 vs 18.62, *P* = 0.01) (Table 5).

**Table 5:** Aortic distensibility in studied patients based on the presence of the metabolic syndrome

	With metabolic syndrome	Without metabolic syndrome	*P* value
AR distensibility			
Kpa^-1^ × 10^-3^	13.36 ± 5.38	17.05 ± 2.31	0.01
AA distensibility			
Kpa^-1^ × 10^-3^	12.48 ± 4.83	16.93 ± 2.66	0.04
DA distensibility			
Kpa^-1^ × 10^-3^	18.03 ± 4.59	18.62 ± 2.97	0.01

## Discussion

In this study, aortic distensibility was significantly lower in the prediabetes group than in the control group. We also found a significant negative correlation between aortic distensibility and the levels of HbA1C. Impaired glucose tolerance alters the mechanical properties of the interstitial tissue of the vascular wall; moreover, it enhances nonenzymatic glycation of proteins. Nonenzymatic glycation leads to the formation of increased collagen crosslinks that result in increased arterial stiffness [10–12].

In the current study, all patients’ ages were between the 3rd and 5th decades to avoid age effects on aortic elastic properties; that is, in individuals ≥ 55 years of age, PP increased more markedly and strain continued to decline, leading to lesser sensitivity of aortic distensibility as a marker of arterial aging beyond this age [13].

The current study showed that BMI and WC were higher in the study group than in the control group. These findings are consistent with the results of Marini et al., who assessed cardiometabolic risk profiles in individuals with prediabetes [12]. In our study, there was no significant difference in systolic blood pressure (SBP) between the study and control groups; on the contrary, diastolic blood pressure (DBP) and pulse pressure were different, which was not consistent with a randomized controlled trial that assessed BP variability in individuals with prediabetes that showed higher SBP and DBP, which may be explained by the nonuse of ambulatory BP monitoring in our study [13].

In the current study, aortic distensibility (AD) values at different locations (aortic root, ascending aorta, and descending aorta) had significant negative correlations with waist circumference, which was consistent with another study that assessed AD in untreated essential hypertension patients, although they had measured aortic PWV with the SphygmoCor VX system for aortic stiffness assessment [14].

Our control group demonstrated AA distensibility of 28.10 ± 8.33 Kpa^-1^ × 10^-3^ (*P* < 0.001) and DA distensibility of 33.08 ± 7.57 Kpa^-1^ × 10^-3^ (*P* < 0.001), which is consistent with the values reported by Redheuil et al. in a cross-sectional randomized controlled trial that assessed distensibility by MRI (21.3 ± 2 Kpa^-1^ × 10^-3,^ *P* < 0.0001) [8].

In our study, aortic distensibility at the aortic root and descending aorta was not significantly correlated with the age of the subjects, AR (*r* = − 0.03, *P* < 0.83), and DA (*r* = − 0.08, *P* < 0.56); however, the distensibility of the ascending aorta had a moderate significant correlation with age, which may be attributed to the higher elastin content of the ascending aorta that may decrease with age [15].

Our results were also consistent with the MESA study that used fasting glucose levels to diagnose glucose intolerance and its effect on proximal thoracic aortic distensibility by MRI, which found that glucose status had no effect on AD in subjects aged > 65 years old, while aortic distensibility values in the age-matched subgroup were similar to our results (2.11 × 10^-3^ mmHg^-1^ for IFG, *P* = 0.01) [15].

The present study showed that patients with metabolic syndrome have lower AD and that waist circumference, BMI, and HbA1C were stronger predictors of worse AD. Metabolic syndrome increases the risk of CV disease in many ways and increases arterial stiffness in all ages [16–18]. The sympathetic nervous system, renin-angiotensin system, inflammatory cytokines, and hyperdynamic circulation play an important pathophysiologic role in metabolic syndrome [19].

Aortic elasticity indices have been linked to the occurrence of CV events and target organ damage in prediabetics, diabetics, and hypertensives, which emphasizes the importance of assessing aortic distensibility (as one of aortic elasticity markers) in these patients [20, 21].

## Conclusions

Individuals with prediabetes and those with metabolic syndrome have worse aortic elastic properties than those with normal glycemic index values. CMR provides a relatively easy and reproducible method for the assessment of local aortic function in comparison to the cumbersome old methods with commonly encountered limitations and pitfalls [22].

## Data Availability

Data including excel sheets, and MRI results are available

## Abbreviations

AA: Ascending aorta
AD: Aortic distensibility
AR: Aortic root
ATP III: Adult treatment panel III
BMI: Body mass index
CBC: Complete blood count
CCAD: Chronic coronary artery disease
CMR: Cardiac magnetic resonance
CV: Cardiovascular
DA: Descending aorta
DBP: Diastolic blood pressure
DM: Diabetes mellitus
ECG: Electrocardiogram
HbA1C: Glycosylated hemoglobin
HDL: High-density lipoproteins
HTN: Hypertension
IDF: International Diabetes Federation
IFG: Impaired fasting glucose
LDL-c: Low-density lipoprotein cholesterol
LV: Left ventricle
LVEF: Left ventricle ejection fraction
MESA study: Multi-ethnic study of atherosclerosis
MRI: Magnetic resonance imaging
PLAX: Parasternal long axis
SBP: Systolic blood pressure
TC: Total cholesterol
TG: Triglycerides
WC: Waist circumference
